# Aspirin Inhibits Carcinogenesis of Intestinal Mucosal Cells in UC Mice Through Inhibiting IL-6/JAK/STAT3 Signaling Pathway and Modulating Apoptosis and Proliferation

**DOI:** 10.5152/tjg.2022.21855

**Published:** 2022-09-01

**Authors:** Yingying Chen, Liying Sun, Dongyue Li, Xunhai Yin, Guoyin Shang, Tiantian Diao, Lijun Shi

**Affiliations:** 1 Department of Gastroenterology, The First Affiliated Hospital of Harbin Medical University, Harbin, China; 2 Department of Pediatrics, The First Affiliated Hospital of Harbin Medical University, Harbin, China

**Keywords:** Aspirin, colorectal cancer (CRC), IL-6/JAK/STAT3 signaling pathway, inflammation–atypical hyperplasia–cancer, ulcerative colitis (UC)

## Abstract

**Background:**

Colorectal cancer is related to ulcerative colitis. This study aimed to investigate the effects of aspirin on non-specific inflammation developing into cancer.

**Methods:**

Ulcerative colitis model was generated by administrating azoxymethane/dextran sulfate sodium to mice. Weight, tumor size/amount, and intestinal mucositis scores were analyzed. Inflammatory cell infiltration and atypical hyperplasia were determined with hematoxylin–eosin staining. Immunohistochemical assay was used to detect the proliferating cell nuclear antigen. Interleukin-6 and interleukin-10 were detected using enzyme-linked immunosorbent assay. Signal transducer and activator of transcription 3, phosphorylated-STAT3, cyclin D1, and suppressor of cytokine signaling 3 were examined with western blotting.

**Results:**

Aspirin remarkably decreased tumor size/amount compared to those of the ulcerative colitis model group (*P* < .05). Interleukin-6 was increased and interleukin-10 was decreased in mice of ulcerative colitis model group compared with the control group (*P* < .05). Aspirin markedly reduced interleukin-6 and enhanced interleukin-10 compared to the ulcerative colitis model group (*P* < .05) induced Azoxymethane/dextran sulfate sodium inflammation (3 weeks) and atypical hyperplasia (8 weeks). Aspirin predominantly inhibited the “inflammation–atypical hyperplasia–cancer” process and alleviated inflammatory cell infiltration of mice in the ulcerative colitis model group. Aspirin promoted apoptosis and alleviated proliferating cell nuclear antigen of atypical hyperplastic intestinal mucosal cells at 8 weeks post-modeling. The expression of phosphorylated-STAT3, signal transducer and activator of transcription 3, cyclin D1, and suppressor of cytokine signaling 3 was significantly increased in mice of ulcerative colitis model group compared to the control group (*P* < .05). Aspirin remarkably decreased phosphorylated-STAT3, signal transducer and activator of transcription, and cyclin D1 expression compared with ulcerative colitis model group (*P* < .05).

**Conclusion:**

Aspirin inhibited carcinogenesis of intestinal mucosal cells in the ulcerative colitis model by inhibiting the interleukin-6/Janus kinase/signal transducer and activator of transcription 3 signaling pathway and promoted apoptosis, thereby suppressing proliferation.

**Main Points**

- Aspirin inhibited tumor growth, reduced interleukin-6 (IL-6) levels, and enhanced interleukin-10 levels in ulcerative colitis (UC) mice.
- Aspirin inhibited the “inflammation–atypical hyperplasia–cancer” process and alleviated inflammatory cell infiltration.
- Aspirin promoted apoptosis and alleviated proliferating cell nuclear antigen expression in atypical hyperplastic intestinal mucosal cells.
- Aspirin inhibited carcinogenesis of intestinal mucosal cells in UC model via inhibiting IL-6/Janus kinase/signal transducer and activator of transcription 3 signaling pathway and promoted apoptosis.

## Introduction

Till now, plenty of studies^1-3^ have proven that inflammation is involved in the pathogenesis of a variety of tumors and plays an important role. A few researches^4,5^ reported that chronic inflammatory sites may be the birthplace of tumors. A previous study^6^ showed that about 25% of cancers are correlated with chronic inflammation; therefore, they have attracted great attention. A few former studies^7,8^ have found that in addition to clear infectious factors, some non-infectious inflammations can also increase the risk of tumorigenesis, such as inflammatory bowel disease (IBD), which may be involved in the occurrence and development of tumors. Additionally, a few studies^9,10^ have also found that colorectal cancer (CRC) is closely related to ulcerative colitis (UC) and Crohn’s disease. However, in the biological process of non-specific inflammation developing into cancer, it is not known which biological reaction occurs, which biological factors play a role, and which related signal pathways are involved in these processes.

Carcinogenesis mechanism of UC is mainly related to recurrence rate, severity, and duration of disease.^11^ Because the long cancerous process of UC, collection of cases usually takes several years or even decades and has adversely affected clinical research on the carcinogenesis mechanism of UC. Therefore, it is necessary to establish an animal model to study the carcinogenesis mechanism of UC. So far, a variety of animal experimental models have been established in the study of UC, but there are only few models that can play a role in studying the inflammation carcinogenesis mechanism.^12^ Among the established models, the most widely used animal model is induced by dextran sulfate sodium (DSS), which can show very similar pathogenesis and clinical characteristics to human UC.^13^ However, the DSS-induced UC model takes a long time to induce intestinal tumors based on the model. In recent years, experts and scholars have conducted in-depth researches. Okayasu et al^14^ proposed for the first time in 1996 that the combination of single-dose mutagen azoxymethane (AOM) and DSS can successfully establish the carcinogenesis model of UC. This model takes only a short time to carcinogenesis and demonstrates the dynamic process of inflammation developing into cancer cells, which is helpful to explore the molecular mechanism of carcinogenesis.

Signal transducer and activator of transcription 3 (STAT3) is one of the members of the STAT signaling pathway family. At present, it is found that STATs family molecules play a role in the transmission of signal pathway and can receive external stimulation signals.^15^ Then, STAT3 is activated by external signals or molecules and plays a role in physiological mechanisms, such as cell proliferation and apoptosis.^16^ The study also found that a variety of cytokines and growth factors can complete signal transduction and have related effects through Janus kinase (JAK)/STATs signal pathway. A recent study^17^ has shown that JAK/STATs signaling pathway plays an important role in the occurrence and development of inflammatory diseases and tumors. However, there are few reports on the dynamic changes of signal molecules in evolution for inflammation atypical hyperplasia carcinogenesis.

This study aimed to investigate the effects of aspirin (ASA) on non-specific inflammation developing into cancer. Meanwhile, the pathogenic process of colonic tissue from normal mucosa to inflammatory mucosa to atypical hyperplasia to carcinogenesis was also explored.

## Materials and Methods

### Animals

A total of 16 specific pathogen-free male Balb/c mice age-matched (6-7 weeks) with a weight of 20 ± 2 g were purchased from Beijing Huafukang Biosci. Co. Ltd. (Beijing, China). The mice were housed in plastic cages under the condition of a 12/12 h cycle of light/dark with controlled humidity and temperature and free access to food and water.

All experiments involving animals were in accordance with the guidelines of the ethical procedures suggested by the Animal Care and Use Committee of our hospital.

### Establishment of Azoxymethane/Dextran Sulfate Sodium Model and Grouping

Azoxymethane/dextran sulfate sodium (AOM/DSS) model was established as described by former studies,^18,19^ with a few modifications. Briefly, mice were weighed and fed adaptively for 1 week. Azoxymethane (10 mg/kg, Cat. No. A5486-25MG, Sigma-Aldrich, St. Louis, Mich, USA) was intraperitoneally injected into mice. One week later, mice were given 3% DSS (molecular weight: 36 000-50 000, Cat. No. 0216011080, MP Biomedicals, Solon, OH, USA) continuously in drinking water for 4 days and then fed with ordinary drinking water for 10 days. The above process is defined as a cycle. After intraperitoneal injection of AOM (10 mg/kg) in the last 2 cycles, 2% DSS was used for 4 days.

The mice were randomly divided into 4 groups, including control group (n = 4), UC model group (n = 4), UC + low-ASA group (n = 4), and UC + high-ASA group (n = 4). Mice in the control group were fed with normal food and water without additional treatments. Mice in the model group were administered with the modeling process as mentioned above. Mice in UC + low-ASA group and UC + high-ASA group were given 200 mg/kg and 400 mg/kg of ASA in their diets, respectively, throughout the present animal experiment. The dosage of aspirin was determined according to a previous study^20^ and the pre-experiments of our team. In the present study, in AOM/DSS-induced CRC mice, ASA was added to the diet of mice from the initial stage of the experiment. Aspirin was added in proportion of 200 mg/kg or 400 mg/kg diet. In order to increase the residence time of ASA in the gastrointestinal tract of mice, ASA was added to the diet instead of direct gavage. At the same time, in order to ensure that each mouse eats the same amount of feed, we ensured that each mouse consumes 2.5 g ASA-containing feed (200 mg/kg or 400 mg/kg, each mouse was fed separately), that is 500 µg or 1000 µg of aspirin.

### Sample Collection

The mice were first anesthetized with sodium pentobarbital at a dosage of 100 mg/kg intraperitoneally, and the eyeball blood was collected after 3 or 8 weeks. A total of 500 μL eyeball blood samples were collected into Eppendorf tube and stored at −80°C until the analyses. All mice were sacrificed after 3 or 8 weeks by cervical dislocation, following the eyeball blood collection. Then, the colonic tissues of mice were isolated and divided into 2 parts. One part was fixed using 4% paraformaldehyde for subsequent histopathological study and hematoxylin–eosin staining and the other part was stored at −20°C for subsequent western blotting analysis. Meanwhile, ulcer, inflammation, mass, tumor size, location, and number were observed and recorded. The tissues from the most serious ulcer, atypical hyperplasia, and tumor were also collected.

### Basic Data Measurement

For the mouse model, basic data including changes in body weight, tumor amounts, tumor size, and intestinal mucositis score were collected. Changes in body weight were measured and determined as described in Capuano et al’s study.^21^ The calculation formula was defined as follows: Delta% = (*W*_f_ − *W*_i_)/*W*_i_ × 100, where “*W*_f_” represents the final weight and “*W*_i_” represents the initial weight. The tumor size was recorded and calculated using the following formula: tumor volume (mm^3^) = (long diameter × short diameter^2^) × 0.52. The intestinal mucositis score was calculated according to a former study.^22^

### Histopathological Determination

For histopathology examination, the paraformaldehyde-treated colonic tissues were dehydrated using gradient alcohol, embedded in paraffin, and cut into 5-μm sections. Then, colonic tissue sections were stained using hematoxylin–eosin and observed under a microscope (Model: AX70, Olympus, Japan). The pathological scores of colitis were evaluated as described in the study by Cooper et al.^23^

### Immunohistochemical Evaluation

For the immunohistochemical evaluation, the paraformaldehyde-treated and paraffin-embedded colonic sections were deparaffinized using xylene and hydrated using gradient alcohol. Sections were treated with 0.2% Triton X-100 for 4 minutes and 0.5% Triton X-100 for 10 minutes to conduct antigen retrieval. Sections were treated with 3% hydrogen peroxide (15 minutes) to inactivate endogenous peroxidase activity and blocked using 6% goat serum in phosphate-buffered solution (PBS, pH 7.4) for 20 minutes at room temperature. Then, sections were incubated using rabbit anti-mouse proliferating cell nuclear antigen (PCNA) (Cat. No. ab92552, Abcam Biotech., Cambridge, Mass, USA) at 4°C overnight. After washing 3 times with PBS (5 minutes per time), sections were incubated with biotinylated goat anti-rabbit antibody (Cat. No. BAF017, RD Systems, Minneapolis, Minn, USA). Finally, sections were visualized with diaminobenzidine (Cat. ZLI-9017, ZSGB Bio., Beijing, China), counterstained with hematoxylin, and observed under a microscope (Model: AX70, Olympus, Japan).

### Enzyme-Linked Immunosorbent Assay

Levels of interleukin-6 (IL-6) and interleukin-10 (IL-10) in the colonic tissues and serum were detected with the commercial Mouse IL-6 ELISA Kit (Cat. No. Ek-M20193) and IL-10 ELISA Kit (Cat. No. Ek-M20153), respectively, as instructed by manufacturer’s protocols (Shanghai Bio. Tech. Co. Ltd. Enzyme Res., Shanghai, China). The enzyme-linked immunosorbent assay (ELISA) 96-well plates were read on an ELISA reader (Model: ELX-800, Biotek Winooski, VT, USA) at 450 nm. The standard curve and optical density data were produced with microplate reader software (version: Gen5 2.0, Biotek).

### Terminal Deoxynucleotidyl Transferase-mediated dUTP Nick-end Labeling

To detect apoptotic cell death in ileum tissues, a terminal deoxynucleotidyl transferase-mediated dUTP nick-end labeling (TUNEL) assay (In Situ Cell Death Detection Kit, Roche, Mannheim, Germany) was performed using TUNEL detection kit, according to manufacturer’s instructions (Cat. No. 11684817910, Roche, Mannheim, Germany). Ileum tissue sections were incubated with proteinase K for 20 minutes at room temperature and washed with PBS. After that, the sections were incubated in terminal deoxynucleotidyl transferase buffer containing fluorescein isothiocyanate-conjugated dUTP at 37°C for 60 minutes. Morphological changes in the nuclei were observed by counterstaining with 2-(4-amidinophenyl)-6-indolecarbamidine dihydrochloride (Beyotime, Shanghai, China). Sections were analyzed under a Nikon fluorescence microscope (Eclipse Ti-SR, Nikon Corporation, Tokyo, Japan).

### Western Blotting Analysis

Total proteins in the colonic tissues (serious ulcer, atypical hyperplasia, or tumor tissues) were separated using radioimmunoprecipitation assay buffer (ApplyGen., Beijing, China). The concentrations of the above-­isolated proteins were determined with a bicinchoninic acid Protein Detection kit (Beyotime Biotech., Shanghai, China) as instructed by the manufacturer. Proteins were separated with 10% sodium dodecyl sulphate-­polyacrylamide gel electrophoresis (Amersham Biosciences, Piscataway, NJ, USA) and electro-­transferred onto the polyvinylidene fluoride (PVDF) membrane (DuPont, Wilmington, Del, USA). Then, the PVDF membrane was washed with the Tris-buffered saline tween 20, blocked with 5% non-fat milk at room temperature for 1 hour, and incubated with mouse STAT3 antibody (Cat. No. MAB1799, RD Systems, Minneapolis, Minn, USA), phospho-STAT3 antibody (Cat. No. AF4607, RD Systems), mouse cyclin D1 antibody (Cat. No. AF4196, RD Systems), mouse suppressor of cytokine signaling 3 (SOCS3) antibody (Cat. No. MAB5696, RD Systems), and mouse glyceraldehyde-3-phosphate dehydrogenase antibody (Cat. No. A01622, GenScript., Nanjing, China) at 4°C overnight. Subsequently, PVDF membrane was washed with PBST and incubated with horseradish peroxidase (HRP)-conjugated goat anti-mouse IgG (Cat. No. 12-349, Sigma-Aldrich, St. Louis, Miss, USA) or HRP-conjugated goat anti-rabbit IgG (Cat. No. 12-348, Sigma-Aldrich), or at room temperature for 1 hour. Eventually, the PVDF membrane was imaged using enhanced chemiluminescence kit (Thermo Scientific Pierce, Rockford, Ill, USA) as per the manufacturer’s protocol.

### Statistical Analysis

Statistical analyses were carried out with Statistical Package for the Social Sciences 19.0 (IBM Corp., Armonk, NY, USA), and the data were defined as mean ± standard deviation (SD). The one-way analysis of variance followed by Tukey’s post hoc test was used for comparing the differences among multiple groups. *P* < .05 was assigned as the statistical difference.

## Results

### Aspirin Inhibited the Tumor Growth in UC Mice

The results showed that mice in the UC model group and the ASA-treated groups had bloody stool or loose stool and weight loss on the sixth day after AOM injection and could basically recover after 14 days. At the end of the third cycle treatment, half the mice in the UC model group and ASA-treated groups demonstrated colon masses. Meanwhile, the evolution process of “inflammation–atypical hyperplasia–cancer” was observed in the colorectal mucosa of the same sample. Most of the above colon masses were located in the distal segment, followed by the middle segment, and not in the anterior segment.

**Figure 1.**
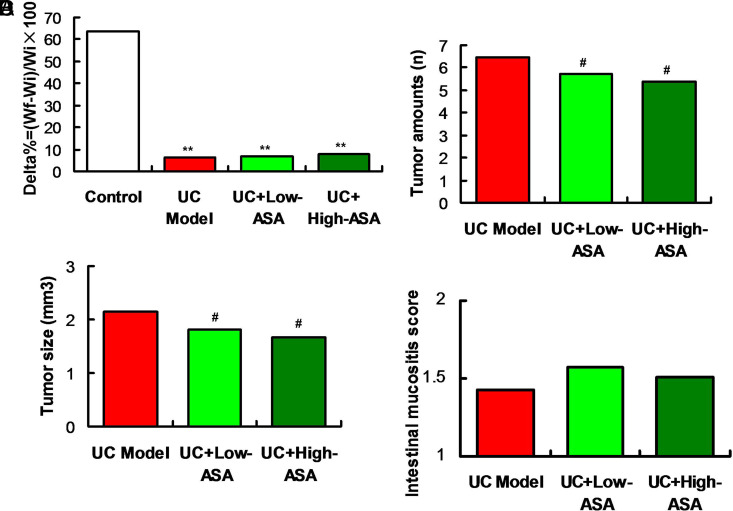
Measurement of the changes of body weight (A), tumor amounts (B), tumor size (C), and intestinal mucositis score (D) of UC mice. n = 4 for each group **P < 0.01 versus control group. ^#^ *P* < .05 versus UC model group. ASA, aspirin; UC, ulcerative colitis.

There was no significant difference in food consumption among the control group, UC model group, UC + low-ASA group, and the UC + high-ASA group. The weight of mice in the UC model group, UC + low-ASA group, and UC + high-ASA group decreased significantly compared with that in control group (Figure 1A, all* P* < .01). However, there was no significant difference in the weight gain among mice in the UC model group and the ASA-treated groups (Figure 1A, *P* > .05). The colorectal tumors appeared in the UC model group, UC + low-ASA group, and UC + high-ASA group. The tumor amounts (Figure 1B) and tumor size (Figure 1C) in UC + low-ASA group and UC + high-ASA group were significantly less (or small) than those in the UC model group (all *P* < .05). However, there were no significant differences in severity of enteritis among mice in the UC model group and ASA-treated groups (Figure 1D, *P* > .05).

### Aspirin Reduced Interleukin-6 and Enhanced Interleukin-10 Levels in UC Mice

**Figure 2.**
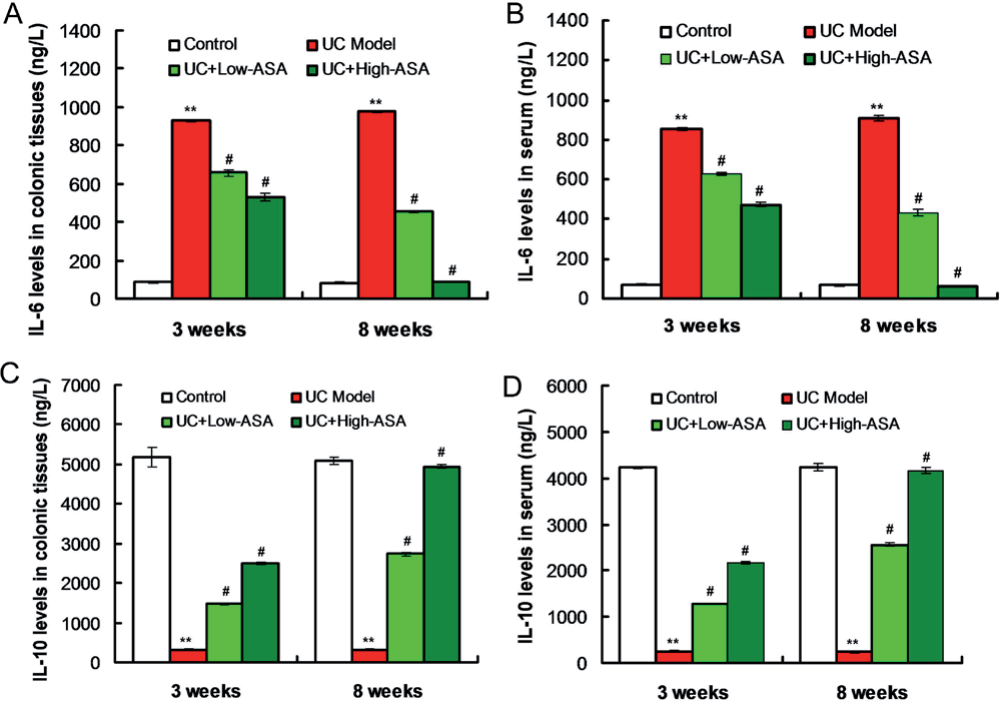
Enzyme-linked immunosorbent assay analyses for detecting IL-6 and IL-10 levels in colonic tissues and serum of UC mice (n = 4 for each group). (A) Interleukin-6 levels in colonic tissues. (B) IL-6 levels in serum. (C) IL-10 levels in colonic tissues. (D) IL-10 levels in serum. **P < .01 versus control group. ^#^ *P* < .05 versus UC model group. ASA, aspirin, UC, ulcerative colitis; IL-6, interleukin-6; IL-10, interleukin-10.

Enzyme-linked immunosorbent assay findings showed that IL-6 levels were significantly increased in the UC model group compared with those in the control group at 3 and 8 weeks post-modeling, in both colonic tissues (Figure 2A) and serum (Figure 2B) of UC mice (all *P* < .01). Aspirin treatments (UC + low-ASA and UC + high-ASA group) remarkably reduced the IL-6 levels compared with those of the UC model group in both colonic tissues (Figure 2A) and serum (Figure 2B) of UC mice (all *P* < .05). Meanwhile, IL-6 levels in UC + high-ASA group were obviously lower compared with those in the UC + low-ASA group (Figure 2A, B, *P* < .05). Moreover, IL-10 levels were remarkably decreased in the UC model group compared with those in the control group in both colonic tissues (Figure 2C) and serum (Figure 2D) of UC mice (all *P* < .01), however, which were blocked (enhanced) by treatments of ASA (UC + low-ASA and UC + high-ASA group) (all *P* < .05). Also, high-dosage ASA (UC + high-ASA) demonstrated more obvious effects on the enhancement of IL-10 levels in both colonic tissues (Figure 2C) and serum (Figure 2D) when compared with those of low-dosage ASA (UC + low-ASA) (all *P* < .05).

### Aspirin Inhibited “Inflammation–Atypical Hyperplasia–Cancer” Process and Alleviated Inflammatory Cell Infiltration

**Figure 3.**
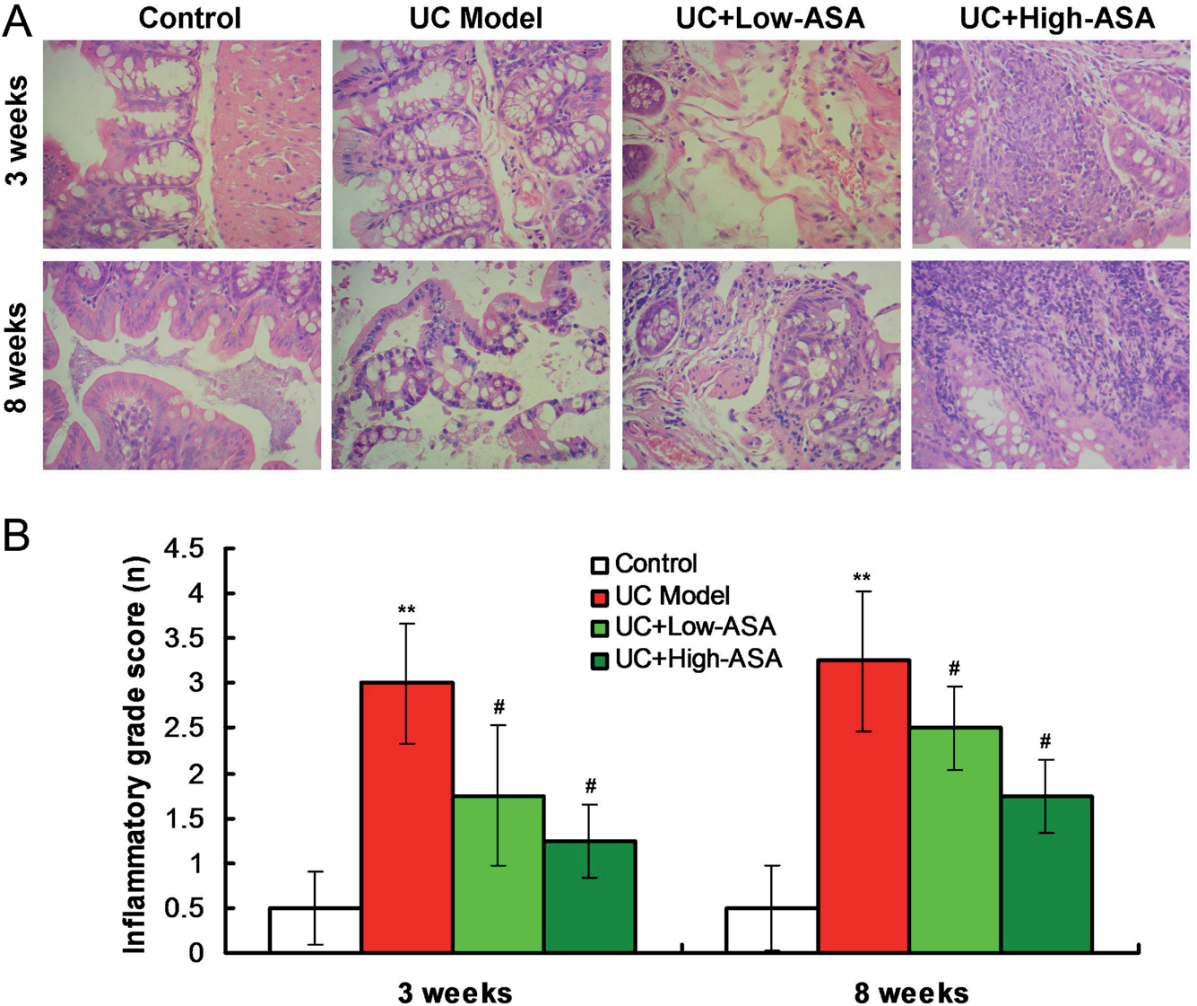
Hematoxylin–eosin staining for “inflammation–atypical hyperplasia–cancer” process and inflammatory cell infiltration (n = 4 for each group). (A) Hematoxylin–eosin staining images for the colonic tissues of UC mice. (B) Statistical analysis for inflammatory grade score. **P < .01 versus control group. ^#^ *P* < .05 versus UC model group. ASA, aspirin; UC, ulcerative colitis.

At 3 weeks post-UC modeling, distal colonic mucosal glands of mice in the UC model group were destroyed in a wide range; some epithelial cells were removed and a large number of inflammatory cells were infiltrated in the mucosal layer (Figure 3A). However, the lesion degree of proximal colonic mucosal glands of mice was mild, the epithelium was complete, the recess structure was normal, a small number of inflammatory cells were infiltrated, and there were no obvious histological changes (Figure 3A). In the control group, the mucosal epithelium was complete, glands were arranged regularly, the structure was complete, and there was no inflammatory cell infiltration (Figure 3A).

At 8 weeks post-UC modeling, mice in the UC model group gradually showed gland hyperplasia, different cell size and morphology, disordered arrangement, loss of polarity, large/deep staining of nucleus, an increased proportion of nucleus to cytoplasm, irregular nuclear shape, increased mitotic image, and other atypical hyperplasia and invasive cancer in varying degrees (Figure 3A). At the same time, the evolution of the “inflammation–atypical hyperplasia–cancer” process was also observed in the colonic mucosa of the same sample (Figure 3A). The cancerous site was mainly located in the distal part of the large intestine. Also, manifestations of atypical hyperplasia and invasive carcinoma of intestinal mucosa were reduced, and the infiltration of inflammatory cells was reduced in mice of both the UC + low-ASA group and the UC + high-ASA group (Figure 3A).

Moreover, according to Cooper inflammation grading criteria, the pathological scores of colitis in the UC model group were predominantly increased compared with those in the control group, at both 3 and 8 weeks post-modeling (Figure 3B, all *P* < .01). However, the ASA treatments significantly decreased the pathological scores of colitis of mice compared with those in the UC model group, both at 3- and 8-weeks post modeling (Figure 3B, *P* < .05). Additionally, mice in the UC + high-ASA group demonstrated markedly lower pathological scores of the colitis compared with mice in UC + low-ASA group (Figure 3B, *P* < .05).

### Aspirin Promoted Apoptosis of Atypical Hyperplastic Intestinal Mucosal Cells

**Figure 4.**
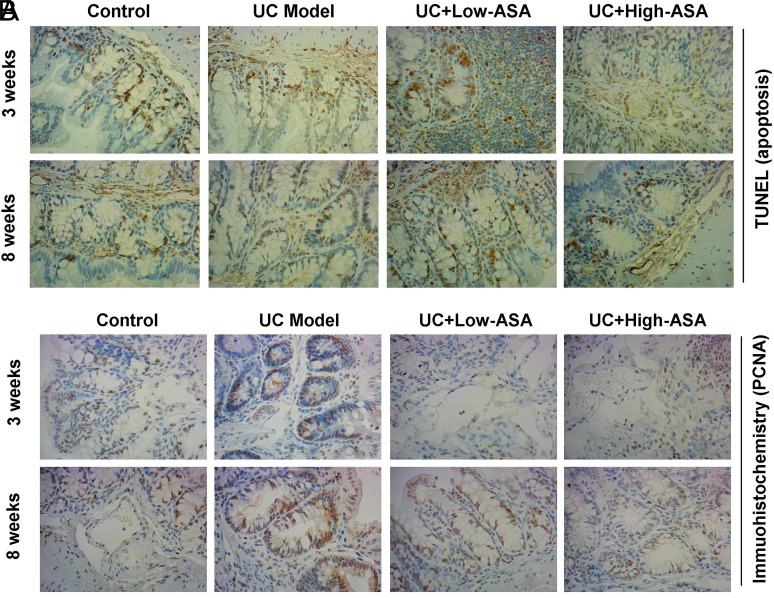
Effects of ASA treatment on apoptosis and PCNA expression in atypical hyperplastic intestinal mucosal cells of UC mice (n = 4 for each group). (A) TUNEL staining for examining apoptosis in atypical hyperplastic intestinal mucosal cells. (B) Immunohistochemical assay for determining PCNA expression in atypical hyperplastic intestinal mucosal cells. ASA, aspirin; PCNA, proliferating cell nuclear antigen; TUNEL, terminal deoxynucleotidyl transferase-mediated dUTP nick-end labeling; UC, ulcerative colitis.

Terminal deoxynucleotidyl transferase-mediated dUTP nick-end labeling findings showed that there were no obvious differences in apoptosis among the UC model group and the ASA treatments groups, except for the control group, during the period of no atypical hyperplasia and tumor inflammation (3 weeks post-modeling) (Figure 4A). However, during the period of atypical hyperplasia and tumor inflammation (8 weeks post-modeling), there was a large area of epithelial cell apoptosis in the atypical proliferative tissues after ASA treatment, when compared with UC model group (Figure 4A). These results suggest that ASA cannot significantly induce apoptosis in non-tumor tissues but significantly promote apoptosis of atypical hyperplastic intestinal mucosal cells.

### Aspirin Alleviated Expression of Proliferating Cell Nuclear Antigen in Atypical Hyperplastic Intestinal Mucosal Cells

At 3 weeks post-UC modeling, the expression of PCNA in intestinal mucosal cells of mice in the UC model group was obviously higher than that of mice in the control group (Figure 4B). The expression of PCNA intestinal mucosal cells of mice in the UC + low-ASA and the UC + high-ASA group was decreased compared with that in the UC group, however without any statistical significance (Figure 4B). At 8 weeks post-UC modeling, atypical hyperplasia and cancer cells appeared in the intestinal mucosal cells. The expression of PCNA in the intestinal mucosal cells of mice in the UC model group was remarkably higher than that of mice in the control group (Figure 4B). At the same time, expression of PCNA was decreased significantly in UC + low-ASA group and the UC + high-ASA group compared with that in the UC model group (Figure 4B).

### Aspirin Inactivated JAK/STAT3 Signaling Pathway

**Figure 5.**
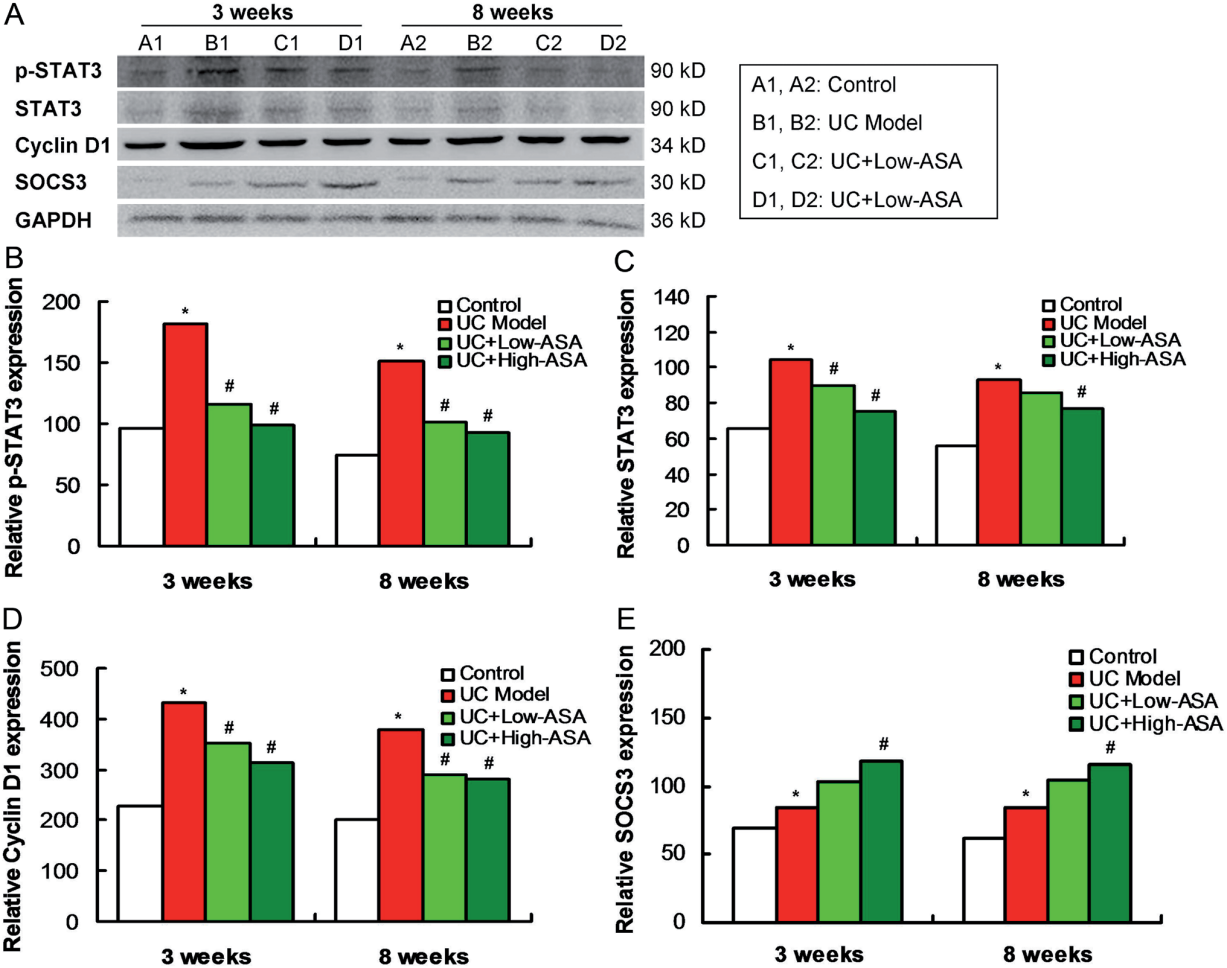
Aspirin modulated the JAK/p-STAT3 signaling pathway in atypical hyperplastic intestinal mucosal cells of UC mice (n = 4 for each group). (A) Western blotting images for JAK/p-STAT3 signaling pathway-associated molecules expression. (B) Statistical analysis and comparison for p-STAT3 expression. (C) Statistical analysis and comparison for STAT3 expression. (D) Statistical analysis and comparison for cyclin D1 expression. (E) Statistical analysis and comparison for SOCS3 expression. *P < .05 versus control group. ^#^ *P* < .05 versus UC model group. JAK, Janus kinase; UC, ulcerative colitis; p-STAT3, phosphorylated-STAT3; STAT3, signal transducer and activator of transcription 3; SOCS3, suppressor of cytokine signaling 3.

In this study, the JAK/STAT3 signaling pathway-associated molecules, including p-STAT3, STAT3, cyclin D1, and SOCS3, were determined using western blotting assay (Figure 5A). The results verified that the expression of p-STAT3 (Figure 5B), STAT3 (Figure 5C), cyclin D1 (Figure 5D), and SOCS3 (Figure 5E) was significantly increased in mice of the UC model group compared with that in mice of the control group (all* P* < .05). Aspirin treatments (UC + low-ASA group and UC + high-ASA group) could remarkably decrease the expression of p-STAT3 (Figure 5B), STAT3 (Figure 5C), and cyclin D1 (Figure 5D), when compared with those of the UC model group (all *P* < .05). However, ASA treatments also increased the expression of SOCS3 compared with that of the UC model group (Figure 5E,* P* < .05). Meanwhile, high-dosage ASA treatment demonstrated more obvious effects on the above molecules expression, however, without significant differences when compared with low-dosage ASA treatment (Figure 5B, C, D, E, *P* > .05). Thus, UC modeling via AOM/DSS induction could activate the JAK/STAT3 signaling pathway, which could be also inactivated by ASA treatment.

## Discussion

Colorectal cancer is a common type of cancer in the existing malignant tumors.^24^ In recent years, the improvement of people’s living standards has also changed the original diet structure in China, where the incidence rate of CRC is high. Studies^25,26^ have shown that patients with IBD are likely to develop CRC after many years of illness, especially patients with UC are more likely to be complicated with colitis-related cancer. A former study^27^ has also confirmed that there is an important relationship between inflammation and tumor and the occurrence of CRC and IBD. Therefore, CRC has become one of the important factors of mortality in IBD patients.^28^

In this study, a small dosage of AOM (10 mg/kg) was injected into the abdominal cavity of mice, and consumption of DSS was random, so as to construct the animal model of UC-associated CRC. Our study detected the cell proliferation-related protein PCNA and core protein p-STAT3 in the STAT3 signaling pathway to explore the pathogenic process of colonic tissue from normal mucosa to inflammatory mucosa to atypical hyperplasia to carcinogenesis, so as to explore the dynamic change rate of JAK/STAT pathway in the process of inflammation to tumor. At the same time, different doses of ASA interfered with JAK/STAT signal pathway to observe whether ASA played a role through this pathway.

Previous studies^19,29^ have confirmed that AOM can induce inflammation-related tumor formation in an experimental animal and has been proven to be a well-known colon carcinogen. The animal model of intestinal chronic inflammation induced by DSS can simulate the process of IBD. The process of IBD greatly increases the incidence of AOM-induced tumors. In this study, the UC animal model was generated by administrating AOM and DSS as our former study reported.^30^ The results indicated that there were many tumors formed with sizes ranging from 1 mm^3^ to 3 mm^3^, which suggests that AOM/DSS treatment induced the tumor formation. While the ASA treatment could significantly decrease tumor amounts and reduce the tumor size, however, the specific mechanisms for the process of “inflammation–atypical hyperplasia–cancer” and effects of ASA have no’t been fully clarified till now.

A former study^31^ showed that an increase of serum IL-6 content in patients with IBD may predict the recurrence of IBD. In intestinal mucosal cells of IBD patients, the abnormally activated STAT3 molecule located in T cells is the core material causing inflammation, and serum IL-6 is a STAT3 activating factor.^32^ A large number of recent experiments show that IL-6/JAK/STAT3 signaling pathway is the key factor causing chronic enteritis.^33^ The content of IL-6 in serum and cancer cells of CRC patients increased significantly, and with the increase of IL-6 content, the tumor volume and diffusion changed significantly.^34^ At the same time, expression of IL-6 is closely related to the treatment of tumor and the survival rate of patients. The IL-6/JAK/STAT3 signaling axis is a core regulatory pathway modulating plenty of gene expression and transcription that play critical functions in the development and progression of cancers.^35^ From the above conclusions, IL-6 is likely to be an important factor for CRC; therefore, the inflammatory factors and inflammations were determined in our study. Enzyme-linked immunosorbent assay findings showed that IL-6 levels were significantly increased and IL-10 levels were significantly decreased in mice of the UC model group, while ASA could reduce IL-6 levels and enhance IL-10 levels of the UC mice. Hematoxylin–eosin staining image also showed that the “inflammation–atypical hyperplasia–cancer” process was observed in the colonic mucosa of the same sample of UC mice. However, ASA treatment inhibited the “inflammation–atypical hyperplasia–cancer” process and alleviated inflammatory cell infiltration. Our results are consistent with the previous studies^36,37^ illustrating that levels of inflammatory-related factors, such as IL-6, would increase in CRC, and tumor recurrence is closely related to the expression of inflammatory factors. Thus, the pathogenesis of CRC is likely caused by the abnormal changes in IL-6/JAK/STAT3 signaling pathway.

At present, it is well known that interfering IL-6/JAK/STAT3 signaling pathway can slow down or inhibit the formation of melanoma, prostate cancer, and gastric tumors.^38^ The reason is that the blocking of this signaling pathway not only inhibits the content of downstream B-cell lymphoma/leukemia-X long molecules but also promotes the normal apoptosis of cells. Meanwhile, during the period of atypical hyperplasia and tumor inflammation (8 weeks post-modeling), the percentages of apoptosis in epithelial cells obviously decreased. Aspirin treatments promoted apoptosis rates when compared with the UC model group. Therefore, ASA could significantly promote apoptosis of atypical hyperplastic intestinal mucosal cells. According to a former study,^39^ PCNA as an important oncogenic transcription biomarker and has been proven to play critical roles during evolution process of “inflammation–atypical hyperplasia–cancer”. In this study, PCNA expressions were increasing obviously from the intestinal mucosal cells (at 3 weeks) to the atypical hyperplastic intestinal mucosal cells (at 8 weeks), suggesting that PCNA levels were increased following with “inflammation–atypical hyperplasia–cancer” process, which is in line with a previous study.^40^ Aspirin could alleviate the expression of PCNA in atypical hyperplastic intestinal mucosal cells. According to TUNEL (apoptosis) findings and PCNA expressions, ASA could exhibit its protective role against the transform from UC (inflammation) to CRC (atypical hyperplasia or cancer) and conduct its apoptosis-inductive effect. Therefore, ASA may be more suitable as one of the combined prevention and treatment for the CRC.

During the development and progression of UC-CRC transformation, there are many evidences for the association among inflammation, atypical hyperplasia, and carcinogenesis. Several inflammation–carcinogenesis-related signaling pathways, such as IL-6/JAK/STAT3, have been proven to involve in the progression of inflammation, epithelial–mesenchymal transition/cell transformation, and malignancy.^41^ Kusaba et al^42^ found that the content of p-STAT3 increased significantly in CRC tissue, and phosphorylated STAT3 (p-STAT3) had a certain impact on tumor growth, metastasis, and invasion. Meanwhile, in IL-6/JAK/STAT3 signaling pathway, the content of cyclin D1 was increased significantly. Therefore, JAK/STAT3 signaling pathway might play a key role in the clinical detection of CRC and it is necessary to explore whether JAK/STAT3 pathway participates in or plays an anti-cancer role. It is reported that IL-6 transduction signal can increase the phosphorylation of STAT3 in tumor cells, which is considered to be a key anti-apoptotic regulator in colitis-related CRC.^43,44^ Our findings showed that p-STAT3 expression was significantly increased in mice of the UC model group compared with that in mice of the control group. Together with the former results of increased levels of IL-6 in UC mice, we could conclude that phosphorylation of STAT3 is helpful in the progression of CRC. Signal transducer and activator of transcription 3 phosphorylation can induce the expression of anti-apoptotic genes (B-cell lymphoma-2 and B-cell lymphoma-XL).^45,46^ Therefore, modulation of the IL-6/STAT3 signaling pathway will become a more targeted treatment for CRC. In our study, ASA treatments remarkably decreased the expression of p-STAT3 in mice when compared with mice of the UC model group. Furthermore, the decreased expression of cyclin D1 is correlated with cell apoptosis.^47^ The present results indicated that cyclin D1 expression in mice of the UC model group was upregulated, which could be inhibited by treating with ASA. Additionally, SOCS3, as an important negative feedback regulator for JAK/STAT signaling pathway,^48^ was also examined in the colonic tissues of mice. As western blotting assay demonstrated, SOCS3 showed the opposite change trend to other molecules in JAK/STAT3 pathway (such as IL-6, p-STAT3), which confirmed that JAK/STAT3 was involved in carcinogenesis. Therefore, our findings revealed that apoptosis in “inflammation–atypical hyperplasia–cancer” process was correlated with IL-6/STAT3 signaling pathway in CRC-associated tumor tissues. On the contrary, ASA could induce apoptosis and inhibit the JAK/STAT3 signaling pathway. Our study further revealed the molecular mechanism of ASA as an anti-cancer drug for the prevention and treatment of CRC and provided a molecular theoretical basis for the use of ASA in treating CRC.

**Figure 6.**
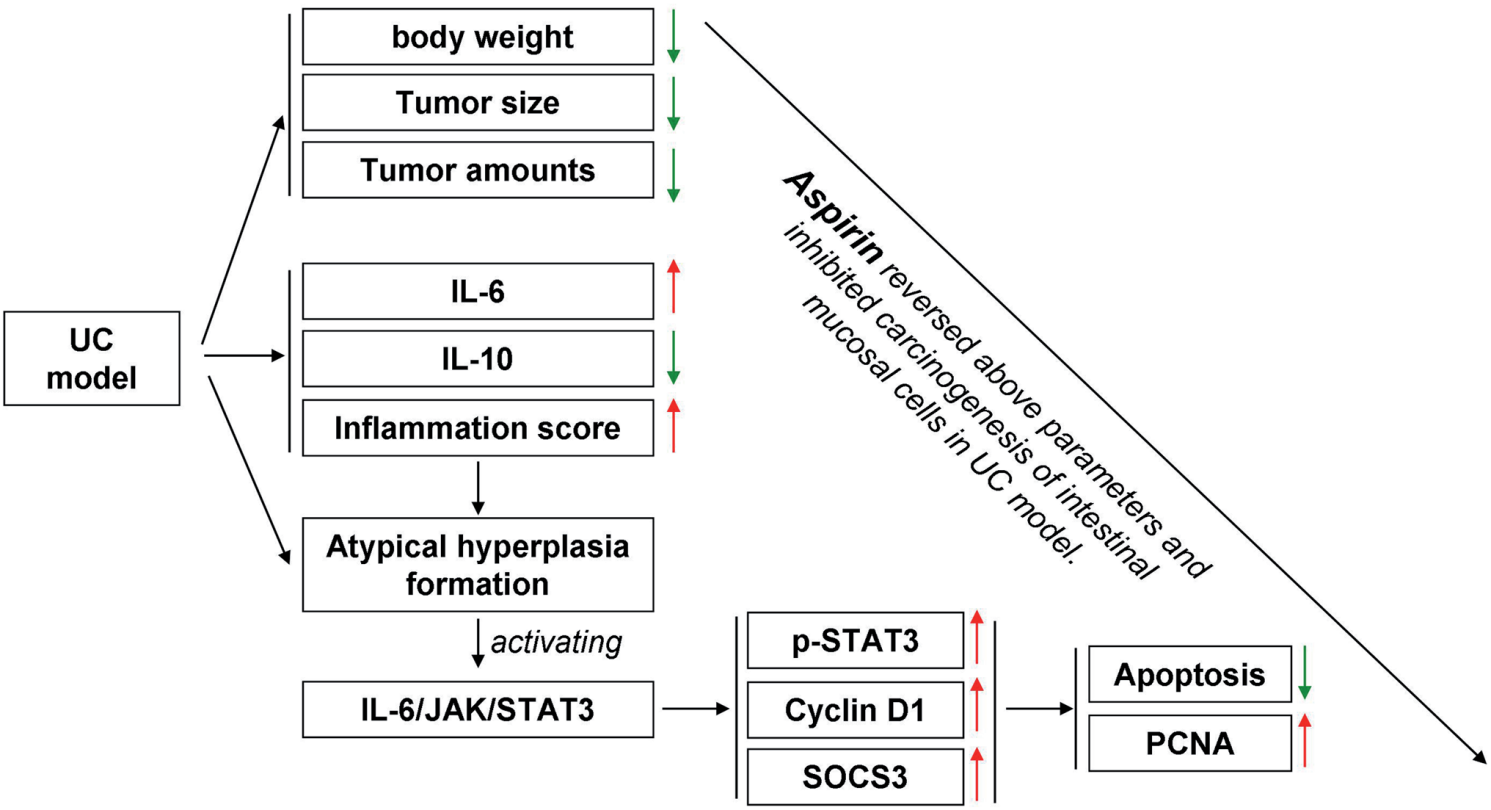
Flow chart for the “inflammation–atypical hyperplasia–cancer” process and effect of ASA in UC mice. ASA, aspirin; UC, ulcerative colitis; IL-6, interleukin-6; IL-10, interleukin-10; JAK, Janus kinase; p-STAT3, phosphorylated-STAT3; STAT3, signal transducer and activator of transcription 3; SOCS3, suppressor of cytokine signaling 3; PCNA, proliferating cell nuclear antigen.

Totally, under pathological conditions, the dynamic balance between cell proliferation, differentiation, and apoptosis is broken.^49^ In this experiment, an animal UC model induced by AOM/DSS was established. In the initial stage, induction of DSS caused acute destruction of the intestinal mucosa and a sharp increase in cell content. It is a kind of pathophysiological compensatory mechanism to repair injury of the intestinal mucosa.^50^ At the same time, JAK/STAT3 signaling pathway is activated due to continuous activation of inflammation, resulting in the persistence of inflammatory response. In this way, the intestinal mucosa was in a state of high-speed reproduction, which increased the probability of genetic factor mutation of mucosal cells, induced the activation of anti-apoptotic factors, resisted the natural death of cells activated under abnormal conditions, and induced inflammation to transform into cancer. Aspirin can promote apoptosis of dysplastic cells in the UC model, block the JAK-STAT3 pathway, and exert an anti-CRC effect (Figure 6).

This study also has a few limitations. First, although it determined the efficacy of ASA against AOM/DSS-induced UC in mice, the present findings should be supported by evidence in other experimental animal models. Second, UC is considered to be a disorder with lots of etiologies; however, the potential or promising pathogenesis of UC has not been fully clarified. Therefore, other signaling pathways involved in UC pathogenesis should be verified in the following study. Third, this study added ASA to the diet but was not given by gavage. It is better to administer ASA by gavage to achieve the standardization of ASA dosage. Additionally, the sample size of the UC mouse model is relatively small in this study.

In conclusion, this study confirmed that the IL-6/JAK/STAT3 signaling pathway is involved in the pathological process of “inflammation–atypical hyperplasia–cancer” in the AOM/DSS-induced UC animal model. Aspirin could downregulate the expression of cyclin D1 and PCNA in atypical hyperplasia, so as to promote apoptosis and suppress proliferation. In summary, ASA inhibits carcinogenesis of the intestinal mucosal cells in the UC model by inhibiting IL-6/JAK/STAT3 signaling pathway and promotes apoptosis, thereby suppressing proliferation.
